# Study on the Degradation of Optical Silicone Exposed to Harsh Environments

**DOI:** 10.3390/ma11081305

**Published:** 2018-07-28

**Authors:** Maryam Yazdan Mehr, Willem van Driel, Francois De Buyl, Kouchi Zhang

**Affiliations:** 1 EEMCS Faculty, Delft University of Technology, Mekelweg 4, 2628 CD Delft, The Netherland; willem.van.driel@signify.com (W.v.D.); g.q.zhan@tudelf.nl (K.Z.); 2 Department of Signify, High Tech Campus, 5600 JW, Eindhoven, The Netherlands; 3 Dow Silicones Belgium sprl, Industrial zone C, 7180 Seneffe, Belgium; francois.debuy@Dow.com; 4 State Key Laboratory of Solid State Lighting, Changzhou 100083, China

**Keywords:** LED, degradation, harsh environment, lighting, silicone, optic

## Abstract

Degradation mechanisms of silicone plates under harsh environment conditions are studied in this investigation. Environmental degradation of silicone free form, used as secondary optics in Light Emitting Diode LED lighting lamps and luminaires or any other applications requiring high quality optics being used, has negative implications for the optical performance. Degradation of silicone plates in harsh environment conditions was studied in salt bath and swimming water environments, using different light radiation and temperatures. Samples were exposed to harsh environment conditions for up to 4 months. Optical and chemical characteristics of exposed plates were studied using an Fourier transform infrared- attenuated total reflection FTIR-ATR spectrometer, an integrated sphere, and a Lambda 950 Ultraviolet-Visible UV-VIS spectrophotometer. Results show that 100 °C salt bath exposure had the most severe degrading effect on the optical characteristic of silicone plates. Increasing exposure time in the salt bath at that high temperature is associated with a significant deterioration of both optical (i.e., light transmission and relative radiant power value) and mechanical properties of silicone samples. On the contrary, silicone plates showed a great degree of stability against light exposure (UV at 360 nm and blue light at 450 nm).

## 1. Introduction

**Figure 1:**
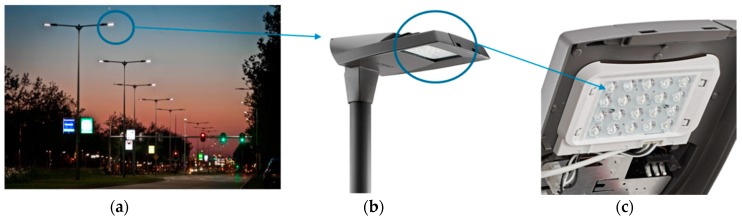
Outdoor applications: street lighting (**a**); outdoor luminaire (**b**); and lens optics (**c**).

In order to control the light beam emitted from an Light Emitting Diode (LED) light source, a secondary optic, such as a free form lens, is used and aligned to the LEDs mounted on the printed circuit board (PCB). This secondary optic can be made of various optically transparent materials, such as glass, polymethylmethacrylate (PMMA), polycarbonate (PC), and silicone. The best material for a given application is highly dependent on technical requirements. Each of these materials has some advantages and disadvantages. Advantages of PMMA is its low cost, easy moldability into any shape, high light transmission, “green” material characteristics, and high UV transmittance. However, PMMA has the disadvantages of yellowing under prolonged UV exposure and higher water absorption. PC material has more benefits than PMMA. PC is harder, has a higher refractive index, is easily molded, has lower water absorption, and higher softening temperature than PMMA (130 °C). The main disadvantages of a PC sample are yellowing under prolonged UV exposure, PC is not eco-friendly, which is not suitable for several biomedical applications, and has a high price. Silicone material is a new kind of optical material that has some benefits over PC and PMMA like higher UV yellowing stability, higher temperature resistance up to 150 °C, low viscosity cure, high flexibility, as well as high impact resistance [1]. Because of its very low surface tension, silicone shows a strong resistance against moisture [1]. Therefore, there is a growing interest in replacing PMMA and PC with silicone in outdoor optical materials to protect components against damage [1,2]. This becomes even more critical in outdoor applications, especially in industrial applications. In our prior study, the photodegradation of polycarbonate under blue light radiation and its effect on optical properties have been described [3]. A limiting controlling factor in the performance and lifetime of lenses in LED-based products is degradation and aging of optical materials during service. This research aims at developing an understanding of the possible degradation of optical and mechanical properties of optical grade silicone plates in harsh environments that may be encountered in outdoor lighting applications. These aforementioned degradations and damages result in a colour shift and lumen decay, with both having serious negative implications for the quality and lifetime of LED lamps or luminaire. In harsh outdoor applications, interactions between temperature, moisture, radiation, and oxidation are inevitable. To our knowledge, there is no report dealing with degradation of silicone lenses in the presence of several degrading factors [4,5]. The information, obtained from the experiments, will be important when it comes to the development of products for harsh outdoor environment applications. Generally, lifetime estimation for LED-based products are typically based on the expected operating hours until light output (e.g., luminous flux) depreciates to 70% of initial levels. The term “lumen maintenance” (lm) is often used to describe the degradation in light output during operation. LM-80 standard is now widely used as an instruction for the luminous flux depreciation test [6]. With the test data provided by LM-80, Lumen degradation estimation method for LED light sources TM-21 standard presents a method for predicting the lumen maintenance of LED light sources beyond 6000 h [6]. A lot of research has been done to model the light output degradation of LED-based products and LED packages [7,8,9,10]. In addition to lumen maintenance, colour shift over service time is another concern for LED manufacturers [11,12,13,14,15,16,17,18]. Both colour shift and lumen depreciation normally take place with relatively slow kinetics. That is why accelerated lifetime testing, followed by extrapolation of data to real service conditions, is needed to estimate the lifetime of LED-based products. Accelerated lifetime testing is essentially based on the exposure of samples to higher levels of degrading factors, i.e., higher-than-normal light intensity, temperature, and humidity [19,20]. Extrapolation of accelerated ageing test results to real service conditions is difficult in case there is a change in the dominant degradation mechanism with changing temperature. Most of the previously done accelerated aging tests are in fact indoor aging tests [3,21,22,23,24,25,26], with temperature and light intensity being most widely used accelerating factors [24,25]. We showed in one of our previous studies that a highly accelerated test set-up (HAST), with 20 times faster kinetics of degradation, can be developed to study the reliability and degradation of LED-based products, using simultaneous temperature and light radiation [25]. Application of combined stresses to do accelerated degradation tests has been reported by Davis et al., in which they applied temperature and humidity at the same time to understand luminaire depreciation [27]. However, to our knowledge, there is no report on degradation and accelerated aging tests of silicone LED lenses in a harsh environment, in which there are other stresses other than temperature and light intensity. In harsh outdoor industrial environments or swimming pool conditions, the package is exposed to UV light, high levels of moisture, and ionic contaminations including exposure to Cl ions. This study is a step forward in making our understanding of the performance and degradation of silicone used as lens in LED-based products in harsh outdoor environments complete. Some examples of outdoor applications are depicted in Figure 1.

## 2. Experimental Procedures

In order to find out the degradation mechanisms of silicone plates under harsh environmental conditions, pure silicone samples of 3 mm thickness, and 3 cm diameter with a refractive index of 1.41 and hardness (Shore A) of 84 were aged under different conditions, given in Table 1:

In order to study the effect of blue light radiation, the high accelerated stress test (HAST, TU-Delft, Delft, The Netherlands) set-up was used to accelerate the aging test up to 10 times [25]. In order to analyze the effect of UV radiation, the UNICORN UV chamber (TU-Delft, Delft, The Netherlands) at room temperature was used. Sample series #4 and #5 were submerged under water boiling at 100 °C with 3 wt % salt, and for series #3, it was underwater in swimming pool water with 3 ppm chloride ions. Samples were exposed to the abovementioned conditions for up to four months. Maximum temperature for the aging of the silicone lens could not be over 120 °C. At temperatures higher than 120 °C, silicone becomes brittle and even fragile. A temperature of 100 °C is high enough to accelerate the ageing in a relatively short time with minimal effects on the mechanical properties of samples. Aging at temperatures lower than 100 °C have been done as well and it did not have any effect on radiation and thermal aging, which has to do with the slow kinetics of reactions at low temperature

Fourier transform infrared (FTIR, TU-Delft, Delft, The Netherlands) spectroscopy was used to study changes in the chemical structure of samples during degradation. Infrared spectra of aged specimens were also recorded using a Perkin–Elmer Spectrum 100 series spectrometer (TU-Delft, Delft, The Netherlands) in the attenuated total reflection (ATR) mode for 200 scans at a resolution of 4 cm^−1^. Infrared spectroscopy (IR) is the spectroscopy that deals with the infrared region of the electromagnetic spectrum. The infrared spectrum of a sample is recorded by passing a beam of infrared light through the sample. When the frequency of the IR is the same as the vibrational frequency of a bond, absorption occurs. The ATR accessories with very small crystals, which is a diamond that is typically about 2 mm across. Diamond is used because it has the best durability and chemical inertness. An IR beam is directed onto an optically dense crystal with a high refractive index at a certain angle. This internal reflectance creates an evanescent wave that extends beyond the surface of the crystal into the sample held in contact with the crystal. In regions of the IR spectrum where the sample absorbs energy, the evanescent wave will be attenuated. The attenuated beam returns to the crystal, then exits the opposite end of the crystal and is directed to the detector in the IR spectrometer. The detector records the attenuated IR beam as an interferogram signal, which can then be used to generate an IR spectrum. A very decisive factor in the accuracy of results is flatness of samples. To obtain the best results with high repeatability, samples must be flat. All silicone samples are fortunately flat and will remain flat during exposure. Samples were first placed on top surface of the crystal. Then the gripper plate was placed on the sample. The pressure applied to the gripper plate was adjusted to ensure that consistent contact was achieved between the crystal and the sample. This was the same for all samples.

Examination of the transmitted light reveals how much energy was absorbed at each frequency (or wavelength).

Chemical analyses were combined with characterization of optical properties of the degraded samples. Integrated sphere and Lambda transmission spectroscopy (TU-Delft, Delft, The Netherlands) were used to study the optical properties of specimens. In order to study changes in the mechanical properties of degraded specimens, a shear punch test was performed on silicone plates, using a DMA Q800 machine (Dow Silicones Belgium, Seneffe, Belgium) with a punch diameter 3 mm.

## 3. Results and Discussion

**Figure 2:**
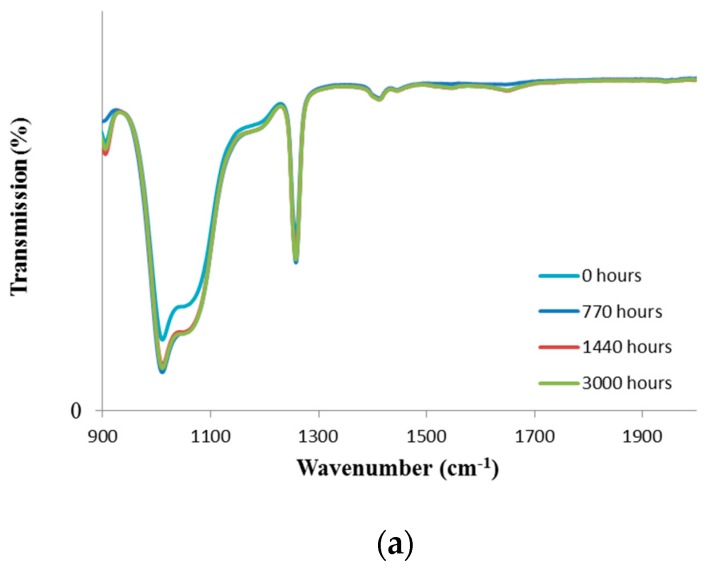

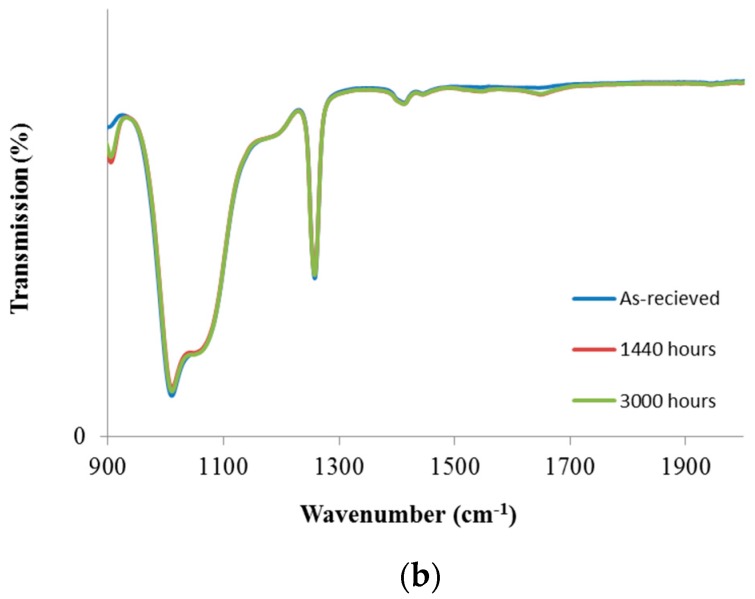
FTIR-ATR spectra of (**a**) UV at 35 °C, and (**b**) blue-light exposed at 100 °C.

Figure 2 shows the effects of blue/UV light radiations on the FTIR spectra of silicone samples. In general, the absorption peak at 1410 cm^−1^ was assigned to the rocking vibration of –CH_2_–. The ones at 1260 and 864 cm^−1^ were assigned to bending and rocking vibrations of Si–CH_3_. The absorption peak at 793 cm^−1^ was due to the coupling of stretching vibration of Si–C and rocking vibration of ACH3. The absorption peaks at 1080 cm^−1^ and 1010 cm^−1^ were attributed to the stretching vibration of Si–O–Si on the backbone of silicone rubbers. It was seen that neither UV nor blue light radiations affected transmission in the FTIR spectra. It appeared that silicone is a stable material under UV light and blue light radiations. As is shown in Figure 2a, there was a small change at 900 and 1680 cm^−1^, but because there was no difference in optical and mechanical properties of aged samples, the changes might have been because of instrument error.

**Figure 3:**
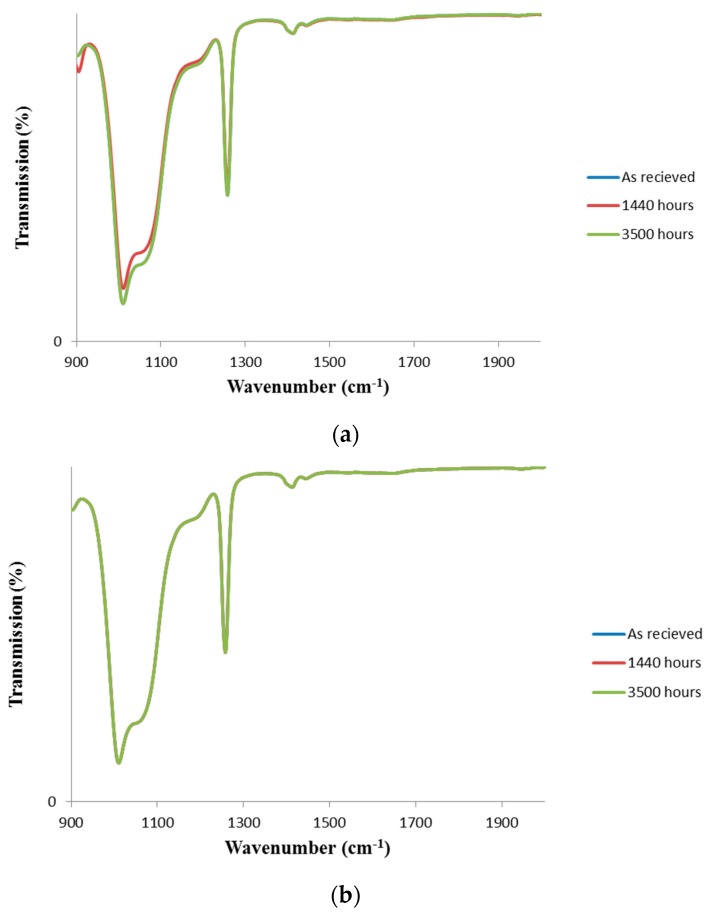
FTIR-ATR spectra of samples exposed to (**a**) swimming pool environment at 100 °C; and (**b**) 100% RH environment at 100 °C.

Figure 3 shows the FTIR spectra of the silicone lens exposed to swimming pool water at 35 °C and to 100% RH environment at 100 °C (sample #4) for up to 3000 h. Again, none of these exposure conditions had any implication for the light transmission in the FTIR spectra.

**Figure 4:**
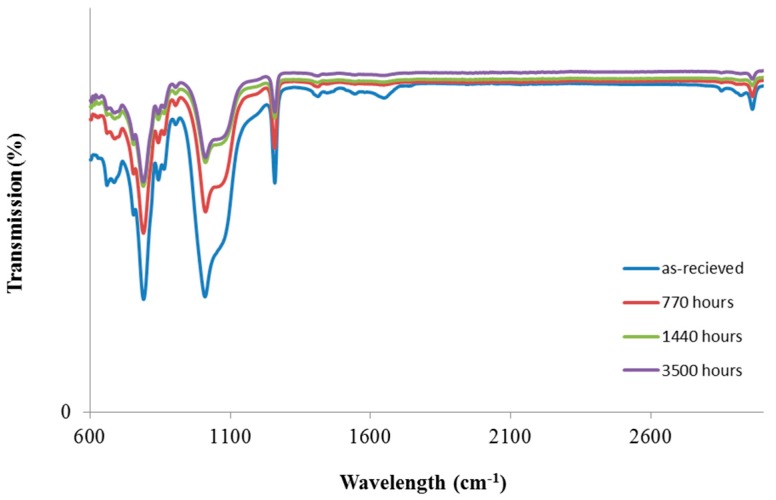
FTIR-ATR spectra of aged Si in a salt batch at 100 °C.

Figure 4 shows the effects of exposure to a saline water environment (sample #5) on the FTIR spectra of silicone samples. Contrary to other exposure conditions, the decrease in the transmission of degraded samples with exposure time in this case is quite significant. This indicates that amongst different possible degrading factors in a harsh environment condition, Cl ions dissolved in water have the most negative contribution to the structural stability of silicone. Cl ions significantly accelerated the kinetics of degradation in silicone samples. Looking at the FTIR spectra, one can conclude that chemical degradation was associated with breaking Si–O–Si bonds and decomposition of methyl groups attached to silicone atoms. Many papers also reported similar results concerning hydrothermal stability of cross-linked silicone rubber [28,29,30,31].

**Figure 5:**
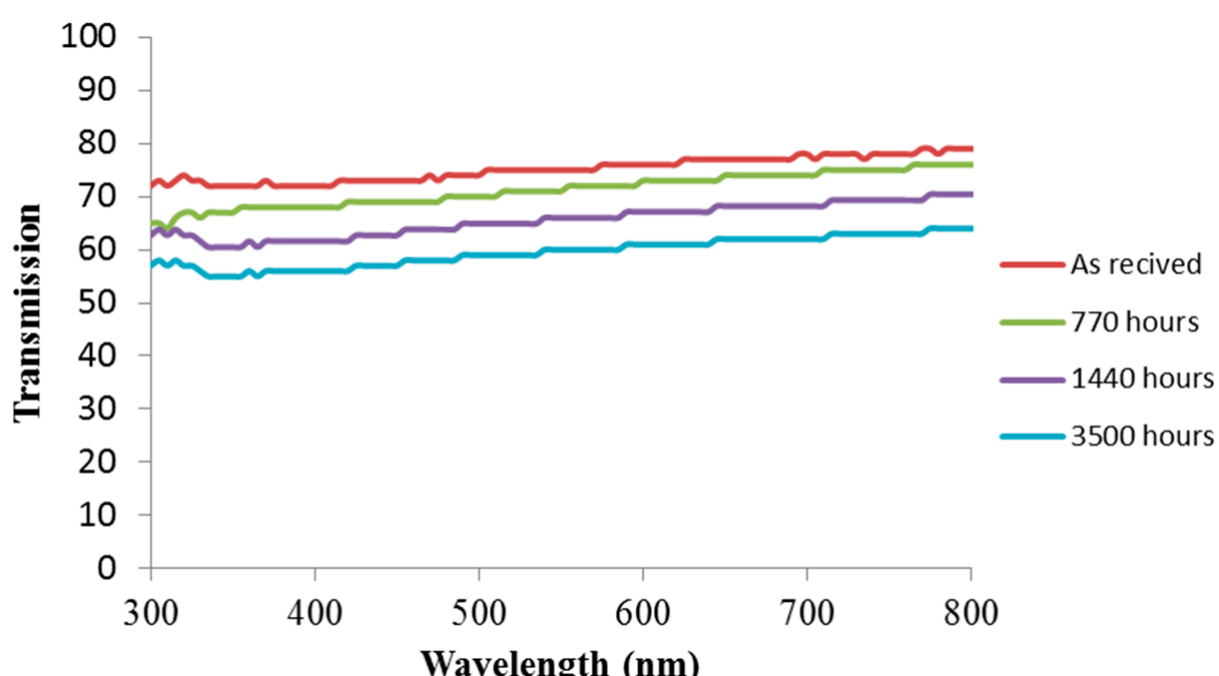
Lambda spectra of 3 mm silicone plates exposed to saline underwater at 100 °C.

Figure 5 shows the UV-visible light transmittance spectra of silicone samples aged under saline water exposure conditions. As expected, the transmission of light decreased in the visible light range with exposure time, which was an indication of deterioration of the quality of silicone plates.

**Figure 6:**
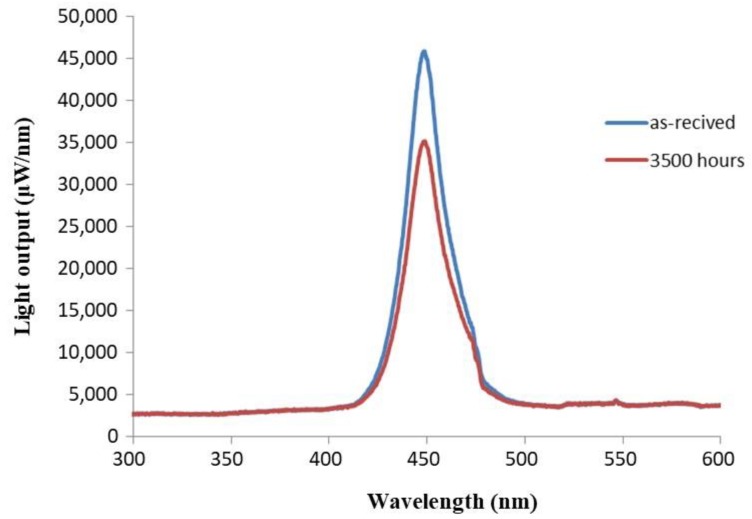
SPD of the saline water-exposed specimen at 100 °C for 3500 h, compared to that of the as-received sample.

**Figure 7:**
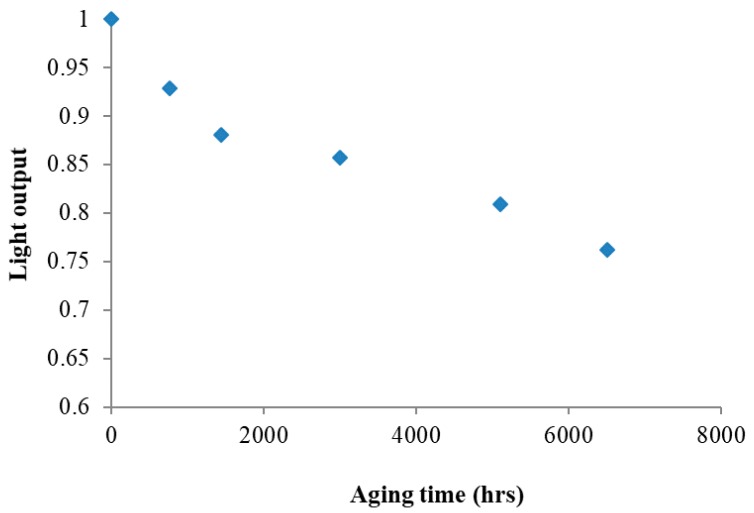
Variation of maximum power intensity at 450 nm with exposure time under a saline water environment condition at 100 °C.

Figure 6 shows the relative luminous intensity (%) versus wavelength (nm) in the spectral power distribution (SPD) spectra of as-received and saline water-exposed samples, showing that the maximum of peak at 450 nm was drastically reduced as a result of degradation. Figure 7 illustrates the effects of aging time on the normalized maximum light output at 450 nm. Interestingly, there was a linear reduction of maximum light output with exposure time.

Table 2 shows the chromaticity values of degraded samples, where these values are average values.

**Figure 8:**
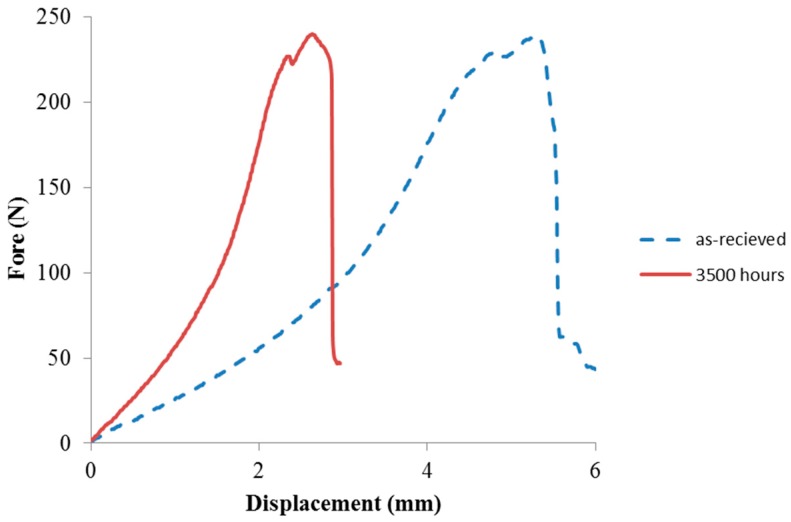
Force displacement curve for aged silicone in a salt bath at 100 ºC.

In order to study changes in the mechanical properties of the degraded specimens in a saline environment, a shear punch test was conducted on as-received and aged specimens with 3500 h of exposure to saline environment. Figure 8 compares force–displacement curves of these two samples, showing that exposure to a saline water environment was accompanied with embrittlement and a decrease in the ductility of specimens.

## 4. Conclusions

The degradation of silicone used in secondary optical designs combined with an LED package under harsh environment conditions was investigated. The effects of UV 360 nm exposure or blue 450 nm light exposure, combined with high temperature (100 °C), water environment (35 and 100 °C), and saline 100 °C water environment on the color shift, transmission, and radiant power intensities of silicone samples were studied. The following conclusions can be drawn:

(1) Silicone samples showed a great degree of stability against light exposures (both UV and blue light). A total of 3500 h of radiation at 100 °C did not result in any change in the optical characteristics of silicone samples.
(2) Exposure to saline and high-temperature environmental conditions had major negative implications for the optical characteristics of the samples. Saline water exposure at 100 °C resulted in a significant reduction in the transmission of samples in the visible light range, reduction in the maximum radiant power at 450 nm, and a change in the color chromaticity values.
(3) Saline water exposure at 100 °C changed the mechanical properties of silicone plates by making them more brittle.

## Figures and Tables

**Table 1:** Experimental exposure conditions.

Sample/Variables	Temperature (°C)	UV Light	Blue Light	Environment
**#1**	35	360 nm	-	Air
**#2**	100	-	450 nm	Air
**#3**	35	-	-	Water (standard swimming pool water)
**#4**	100	-	-	Water (100% RH)
**#5**	100	-	-	Water (100% RH, 3 wt % Salt)

**Table 2:** Variation of chromaticity values with exposure time in saline water-exposed specimens.

Samples	x	y	z
As-received	0.236	0.145	0.619
770 h	0.241	0.151	0.610
1440 h	0.246	0.161	0.573
3500 h	0.251	0.167	0.582
